# Supplementary figures and images for: Effect of physicochemical parameters on the stability and activity of garlic alliinase and its use for in-situ allicin synthesis
Source: PLoS One. 2021 Mar 19;16(3):e0248878. doi: 10.1371/journal.pone.0248878 (PMC7978267; doi:10.1371/journal.pone.0248878)

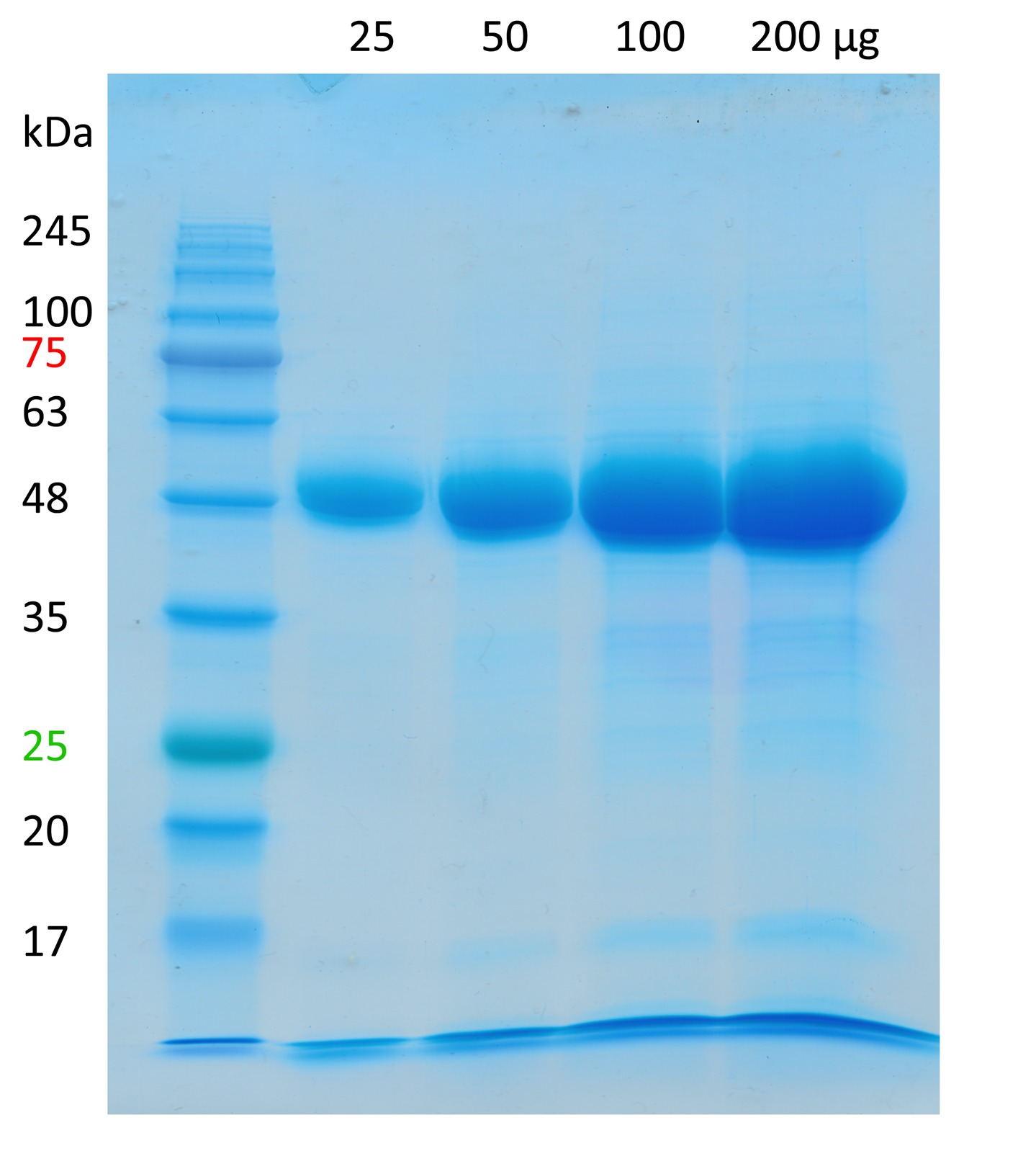

Supplement: S1 Raw images — (TIF) [file pone.0248878.s001.tif]
